# Describing and analysing primary health care system support for chronic illness care in Indigenous communities in Australia's Northern Territory – use of the Chronic Care Model

**DOI:** 10.1186/1472-6963-8-112

**Published:** 2008-05-28

**Authors:** Damin Si, Ross Bailie, Joan Cunningham, Gary Robinson, Michelle Dowden, Allison Stewart, Christine Connors, Tarun Weeramanthri

**Affiliations:** 1 Menzies School of Health Research, Institute of Advanced Studies, Charles Darwin University, Darwin, NT, Australia; 2 School for Social and Policy Research, Institute of Advanced Studies, Charles Darwin University, Darwin, NT, Australia; 3 Northern Territory Department of Health and Community Services, Darwin, NT, Australia

## Abstract

**Background:**

Indigenous Australians experience disproportionately high prevalence of, and morbidity and mortality from chronic illness such as diabetes, renal disease and cardiovascular disease. Improving the understanding of how Indigenous primary care systems are organised to deliver chronic illness care will inform efforts to improve the quality of care for Indigenous people.

**Methods:**

This cross-sectional study was conducted in 12 Indigenous communities in Australia's Northern Territory. Using the Chronic Care Model as a framework, we carried out a mail-out survey to collect information on material, financial and human resources relating to chronic illness care in participating health centres. Follow up face-to-face interviews with health centre staff were conducted to identify successes and difficulties in the systems in relation to providing chronic illness care to community members.

**Results:**

Participating health centres had distinct areas of strength and weakness in each component of systems: 1) organisational influence – strengthened by inclusion of chronic illness goals in business plans, appointment of designated chronic disease coordinators and introduction of external clinical audits, but weakened by lack of training in disease prevention and health promotion and limited access to Medicare funding; 2) community linkages – facilitated by working together with community organisations (e.g. local stores) and running community-based programs (e.g. "health week"), but detracted by a shortage of staff especially of Aboriginal health workers working in the community; 3) self management – promoted through patient education and goal setting with clients, but impeded by limited focus on family and community-based activities due to understaffing; 4) decision support – facilitated by distribution of clinical guidelines and their integration with daily care, but limited by inadequate access to and support from specialists; 5) delivery system design – strengthened by provision of transport for clients to health centres, separate men's and women's clinic rooms, specific roles of primary care team members in relation to chronic illness care, effective teamwork, and functional pathology and pharmacy systems, but weakened by staff shortage (particularly doctors and Aboriginal health workers) and high staff turnover; and 6) clinical information systems – facilitated by wide adoption of computerised information systems, but weakened by the systems' complexity and lack of IT maintenance and upgrade support.

**Conclusion:**

Using concrete examples, this study translates the concept of the Chronic Care Model (and associated systems view) into practical application in Australian Indigenous primary care settings. This approach proved to be useful in understanding the quality of primary care systems for prevention and management of chronic illness. Further refinement of the systems should focus on both increasing human and financial resources and improving management practice.

## Background

Indigenous Australians experience disproportionately high prevalence of, and morbidity and mortality from chronic illness such as diabetes, renal disease and cardiovascular disease. For example, the estimated prevalence of diabetes among Indigenous adults is between 10% to 30%, 2–4 times higher than that of non-Indigenous Australians [1]. Hospitalisation rates for diabetes were 10–15 times higher when compared with the total Australian population [2]. The death rate associated with diabetes for 35–54 year old Indigenous people was 27–35 times that of their non-Indigenous counterparts [3]. The incidence rate of end stage renal disease for Indigenous Australians (779 per million) was 9 times the rate for non-Indigenous Australians (86 per million) [4]. The death rate from chronic renal disease was 10 times higher for Indigenous people than for non-Indigenous people [5]. These national statistics indicate that effective management of chronic illness among Indigenous people should be a high priority primary care service in order to slow the progression of disease and prevent or delay related complications.

While mainstream Australians access primary care through a universal system of general practice funded through Medicare [6], primary care systems for Indigenous people are more complex, with three major services sectors: Indigenous community controlled services, state and territory funded/operated services, and general practices [7]. Indigenous people's access to general practice is limited, particularly in Australia's Northern Territory (NT) where 70% of its Indigenous people live in rural and remote communities where there are few GPs [8]. Therefore, health centres located in Indigenous communities are at the forefront in providing primary health care to the majority of Indigenous people living in the NT.

Many Indigenous community health centres, whether community controlled or state and territory funded, are overwhelmed by providing 'sickness care' for people who are acutely unwell. The focus on acute care services is a result of the high rates of illness in Indigenous communities, combined with limited staff numbers and high staff turnover [9]. While acute care is an ongoing necessity, Indigenous community health centres must at the same time provide effective care for people with chronic illness.

In an international context, the Chronic Care Model (CCM) [10] has gained popularity in recent years as a conceptual model for understanding systems to support chronic illness care and guiding organisational development. It has been adopted and expanded by the WHO to develop an Innovative Care for Chronic Conditions Framework for policy development and system redesign in global contexts [11]. However, clinical training has generally not paid adequate attention to primary care systems, and systems concepts and the value of assessment of the quality of systems may not be fully appreciated by health teams.

Informed by the CCM framework and modern Continuous Quality Improvement theory [12], we conducted a five year quality improvement study (2002–2006), the Audit and Best-practice for Chronic Disease (ABCD) project in Indigenous community health centres in the NT, aiming to support health centre staff to improve their primary care systems for chronic illness care and preventive care. The relationship between the level of health centre system development and quality of diabetes care [13], and the impact of intervention on prevention and management of chronic illness have been reported elsewhere [14,15]. In this paper we report on a practical application of the Chronic Care Model to describe the extent to which Indigenous community health centre systems support chronic illness care and to identify strengths, weaknesses, barriers, and opportunities within these systems.

## Methods

### Study setting and selection of participating health centres

This study was conducted in the Top End of the Northern Territory, an area occupying 522,561 square kilometres, with an estimated resident population of 153,687 in 2003 [8]. In total, there were 53 community health centres located in the Top End. We purposively selected 12 centres for this study to reflect the diversity of health centres in the region in terms of governance models, remoteness, and community sizes. A written participation agreement was signed between the health centre management, its governing body and the Menzies School of Health Research (with which the researchers are affiliated). The study was approved by the Human Research Ethics Committee of the NT Department of Health and Community Services and Menzies School of Health Research, and by its Indigenous health research sub-committee.

Characteristics of the 12 Indigenous community health centres are summarised in Table 1. Compared with all health centres in the Top End, health centres funded/operated by NT government were under-represented in the sample, and health centres with medium-sized populations (500–999) were over-represented. While not a representative sample, these participating health centres reflect the diversity of health centres in the Top End.

**Table 1:** Characteristics of participating community health centres compared to all health centres in the Top End of Northern Territory, Australia

Characteristics	Participating health centres (N = 12)		All health centres (N = 53)	
	n	%	n	%
Health service models				
Indigenous community controlled*	2	17%	4	7%
NT government funded/operated	4	33%	38	72%
Health Board managed^†^	6	50%	11	21%
Sizes of populations served				
< 500	5	42%	27	51%
500–999	5	42%	10	18%
≥ 1,000	2	17%	16	31%
Access to the community				
All year by road	4	33%	18	34%
Part year by road^‡^	6	50%	26	49%
All year by air or sea (islands)	2	17%	9	17%
Kilometres to the nearest hospital				
< 20 km by road	3	25%	6	11%
20–100 km by road	2	17%	6	11%
101–300 km by road	2	17%	18	34%
301–600 km by road	3	25%	14	27%
By air (islands)	2	17%	9	17%

* Refers to health centres legally incorporated and governed by a board elected by the Indigenous community. Most of their funds are from the Commonwealth government through the Office of Aboriginal and Torres Strait Islander Health in the Department of Health and Ageing.

† Refers to a model established through the NT Aboriginal Coordinated Care Trials, in which Commonwealth and NT health funds were provided to an incorporated health board that purchased health services for their community.

‡ Road is often cut off by flood in the wet season (between December and April).

### Measurement and data collection

During 2002–2003, data on primary health care centre systems were collected in 12 community heath centres through three mechanisms: 1) the mail-out system survey; 2) on-site interviews; and 3) the use of the Assessment of Chronic Illness Care (ACIC) scale. The ACIC scale, originally developed by the MacColl Institute for Healthcare Innovation [16], was adapted to assess the level of system development for chronic illness care. Data collection methods for and results of ACIC assessment have been reported previously [13]. The mail-our system survey and on-site interviews are outlined below:

#### Mail-out system survey

A questionnaire was developed for the system survey, aiming to collect information regarding material and financial resources (such as facilities, equipment and funding) and human resources relating to chronic illness care at participating health centres. The questionnaire contained six sections which are consistent with the six system components in the Chronic Care Model).)[10]. Contents of each section are summarised in Table 2. The survey questionnaire was mailed to health centres and completed by the service managers. Information gathered provided the researchers with a general picture of resources in health centres relating to chronic illness care, and also guided subsequent on-site interviews with health staff.

**Table 2:** Contents of Mail-out System Survey questionnaire

Section in the questionnaire	Summary of contents
Organisational influence	AGPAL* accreditation status, discrete funding for chronic illness care, claim for Enhanced Primary Care (EPC) items through Medicare, use of business plan/performance indicators.
Community linkages	Chronic disease related programs running in the community, partnership with other community organisations (through what kind of project or activity), networking with outside organisations.
Self-management support	Use of peer/group education sessions, use of interpreters, teaching aids/resources (videos, posters, models, illustrations, and pamphlets), self-care facilities (weighing scales for the public).
Clinical decision support	Use of best practice guidelines
Delivery system design	Numbers of staff (nurses, Aboriginal health workers, general practitioners, district medical officers, administration/support personnel), gender composition, Indigenous status of staff, duration of employment, years in Indigenous health; details of visiting services (types, frequencies, and adequacy); staff shortage; availability of relief staff; roles of health care team members in relation to chronic illness care; and pharmacy systems.
Clinical information systems	Types of disease register/recall and reminder systems used, software used, computer training.

* Australian General Practice Accreditation Limited.

#### On-site interviews

Face-to-face group interviews were carried out at each participating health centre, attended by service managers, nurses, Aboriginal health workers, and doctors when available. The study was explained to interviewees and verbal consent to their participation, including consent to tape recording of interviews in some cases, was obtained. We took written notes for all interviews. Health centre staff were encouraged to comment on main successes and difficulties in provision of care to the chronically ill.

### Data analysis

Proportions were used to summarise quantitative data as appropriate. Qualitative data were collated using a SWOT (Strengths, Weaknesses, Opportunities, and Threats) analysis [17]. Strengths and weaknesses were defined as being internal to the organisation and opportunities and threats as external. The qualitative data comprised health centre staff comments on their systems. We extracted typical examples from these comments and classified them as strengths, weaknesses, opportunities, or threats for each component of health centre systems.

In addition, an analysis of staff to population ratios was employed to assess the adequacy of human resources for health centres. The actual staff (Aboriginal Health Workers [AHWs], nurses and doctors) to population ratios were compared with an ideal standard of staff to population ratios (Table 3), as developed by the Top End Aboriginal Health Planning Study [18].

**Table 3:** Ideal standard of health service staff to population ratios by community size [18]

Population range	Ideal staff: population ratios		
	
	**AHWs**	**Nurses**	**Doctors**
> 3,000	1:350	1:500	1:1,000
1,300 – 2,999	1:250	1:450	1:1,000
800 – 1,299	1:200	1:300	1:800
400 – 799	1:100	1:200	1:600
250 – 399	1:75	1:200	1:400
75 – 249	1:75	1:150	1:400
< 75	1:50	1:150	1:400

## Results

Results are presented in two parts. The first part is an illustration of the anatomy of a health centre system, showing how components of the Chronic Care Model manifest themselves in a community health centre (a case study). The second part provides in-depth description of each system component in terms of material, financial, and human resources. Likewise, strengths and weaknesses of systems are exemplified using qualitative data.

### Anatomy of a health centre system – a case study

**Figure 1:**
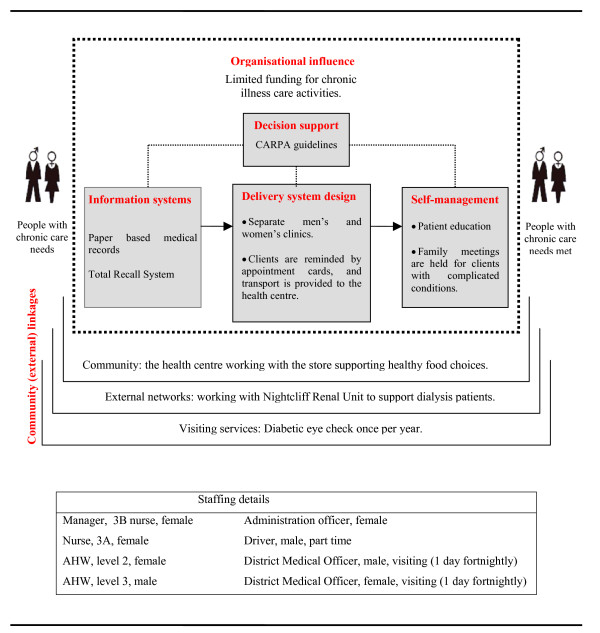
The anatomy of a community health centre system: a case example.

Figure 1 uses a Chronic Care Model framework to illustrate the system anatomy of one participating community health centre. The health centre serves approximately 850 residents living in the community, of whom 29 have a diagnosis of type 2 diabetes. Facilities and activities related to diabetes care can be delineated in six aspects as follows:

#### Clinical information systems

comprise paper-based medical records and a recall system. Medical records are alphabetically arranged in filing cabinets. The Total Recall System, a paper-based recall system developed by the NT Department of Health and Community Services [19], is used in the health centre. The Total Recall System contains two cards and two lists related to chronic illness care: 1) each patient has a *chronic disease card *indicating the chronic diseases the person has and which months checkups are due from January to December; 2) the *visiting specialist/health program list *includes the dates the specialists or health programs are visiting, and the names of people who need these services; 3) the *monthly work list *is generated by a nurse at the beginning of the month by checking the chronic disease cards and the visiting specialist/health program list, and by writing down the names of every person who needs checkups in that month and the scheduled check date for each person; and 4) *appointment cards *are written out each month from the monthly work list.

#### Delivery system design

The number and composition of staff are shown in Figure 1. Clients usually visit the clinic on a "walk-in" basis. In order to encourage clinic attendance, the health centre driver delivers the appointment cards to clients and also provides transport for clients to attend the clinic. The health centre has separate men's and women's clinics for service delivery. A female doctor visits the health centre on Tuesday every second week and a male doctor on Wednesday every second week. The manager coordinates and manages service delivery with assistance from a registered nurse. AHWs are the first point of contact with patients in the health centre. In relation to chronic illness care, nurses and AHWs provide a range of services including basic measurements, ordering lab investigation, taking samples, filling dosettes and dispersing medications to clients. Patients are referred to the doctor on doctor day if necessary. Provision of clinical care is perceived as a team effort, with positive communication and coordination between health practitioners. For example, there is an informal meeting in the morning to talk about the work for the day, who is going where and visitors – who is coming and why.

#### Self-management

has been supported through patient education and family group meetings, however, the latter activities are only for clients with relatively complicated conditions (e.g. patients requiring renal dialysis) and generally not for newly diagnosed chronic disease clients. Setting goals (e.g. for weight control) with clients is used sometimes, but is not a standard practice for all patients.

#### Decision support

The CARPA standard treatment manual [20], developed by the Central Australian Rural Practitioner Association, is the basis for best practice in the health centre and has been used in daily care. For example, the nurse who manages the Total Recall System uses the CARPA guidelines to decide what kinds (and frequencies) of checkups and screening services should be marked in patients' chronic disease cards. With regard to patient self-management, the guidelines are used as the key reference for patient education.

#### Organisational influence

There is a lack of funding to support activities related to chronic illness care. There is no claiming for providing regular checkups for people with chronic disease through Medicare (item number 720) and for holding case conferences (item numbers 740–773). The District Medical Offices are considering claiming these items but the concept is new to them. The health centre manager commented that if there was funding to support an extra position it would reduce the pressure of acute care demands, allowing staff to work outside the clinic more frequently to do health education and promotion with individuals, families and groups, and to facilitate the development of community partnerships with the aim to improve chronic illness care.

#### Community (external) linkages

The health centre forms partnerships with other community organisations (e.g. the community store, the local school) to promote a healthy lifestyle. Linkages between the health centre and external service providers facilitate the use of visiting services at the community.

Through the above examples, the six system components of the Chronic Care Model are shown to be useful in portraying a community health centre's "anatomy" – the set of parts that come together to form the health centre "organism". In the following section we report on collated system information for all participating health centres.

### Description and analysis of health centre system components

#### Organisational influence

Five of twelve health centres included chronic illness care goals in their business plan and the same five had designated staff members (chronic disease coordinators) who were responsible for coordinating chronic illness care (Table 4). Four health centres also received dedicated funding for chronic illness care. Shortage of equipment and lack of training in disease prevention and health promotion were reported by most health centres, and were considered as weaknesses of organisations in addressing chronic illness care (Table 5). While six health centres reported use of Enhanced Primary Care Medicare claims to encourage chronic illness care planning, some staff commented that the incentive effect may be limited due to the returned money not coming back into the budget of that health centre. On the other hand, introduction of public health nurses to conduct external clinical audits at health centres, recently provided by the NT Department of Health and Community Services, was perceived by health service staff as a positive strategy for quality improvement in chronic illness care.

**Table 4:** Organisational influence in health centres (N = 12): resources and management procedures

Resources and management	Number of health centres	%
Business plan containing chronic illness care goals	5	42%
Receive allocated funding for chronic illness care	4	33%
Designated chronic disease coordinator on site	5	42%
Shortage of chronic illness care equipment	9	75%
Lack of clinical training in chronic diseases affecting performance	6	50%
Lack of training in prevention and health promotion affecting performance	10	83%
Claiming for Enhanced Primary Care Medical items (care plans and case conferences)	6	50%
AGPAL* accreditation status		
Currently accredited	6	50%
Scheduled for accreditation	1	8%
No accreditation	5	42%

* Australian General Practice Accreditation Limited.

**Table 5:** Examples of comments on organisational influence

Organisational influence	
**Weaknesses/threats (negative)**	**Strengths/opportunities (positive)**

"Staff come out to the bush and put their career on hold because in this job there is no support for formal training. It seems that when resources are short training is one of the first things to be struck off the list".	"The health centre has two staff members who coordinate the chronic disease care, and their designated time working on chronic disease is 40%".
"There is a shortage of equipment for chronic illness care. Have one of everything but not enough for good service – have to carry from room to room. Do not have enough of everything – need more sphygmomanometers, otoscopes, a BSL machine, scales, and more of the basics".	"Public health nurse comes and does an external audit. This is good, encourages performance and is educative. If you do audits yourself it is easier to dismiss or overlook things. If the audit is external then things are called to our attention".
"A nurse has been trained to get Medicare claims but misses a lot. Hard work and onus on the doctor. Money goes elsewhere. Medicare claim opportunities are being missed".	

#### Community linkages

Some health centres worked together with other organisations and ran community-based programs such as "healthy food choices" at the local store, "health booth", "tobacco prevention week", and "health week" (see Table 6). However, all health service staff reported that acute care demands often prevented the development of community relationships that may have improved chronic care. Other barriers reported by health centres in developing community (external) linkages included shortage of resources (eg vehicles for transport), under-recognition of the worth and benefits of staff working out in the community, and lack of effective communication networks and support arrangements with other organisations.

**Table 6:** Examples of comments on community linkages

Community linkages	
**Weaknesses/threats (negative)**	**Strengths/opportunities (positive)**

The health centre staff spent all of their time within the clinic and there had been no one working in the community for the last 6 months. Having no access to a car was identified as a problem.	The community store supports the healthy food choices via labelling of shelves and using shelf talkers, and meets with the health centre staff fairly regularly to plan. To promote diabetes awareness the store manager has prepared a set of diabetes guidelines for community stores called "No Cry Diabetes".
The health centre has a large numbers of AHWs, whose work includes community visits – but the extent and level of activities is not known. It appears as though much of the work done out in the community by the AHWs is not recorded, therefore may go unrecognised and may be undervalued.	In collaboration with the community store, the health centre set up a health booth outside the store, providing education and well people's screening for passers by and people going shopping. "Do blood pressure, BSL, cholesterol, and BMI. Target groups are men above 45 years and obese. If people have a problem or are sick then give them information and suggest that they make an appointment".
There are budgetary problems in the health centre to cover costs of external services needed to train staff or provide services.	Tiwi for Life program is funded by the Health Board (governing body of the health centre) and has focus of prevention and health promotion in the community. The program implements a range of activities, such as sports days and tobacco prevention week. Communication between the program and the health centre is perceived as good. The health centre has also developed networking with other organisations. For example, a Health Week is held once per year in the clinic in partnership with Tiwi for Life, Department of Health and Community Services, Diabetes Australia, and Council for Aboriginal Alcohol Program Services.
Sometimes there is a lack of communication between the health centre and the external services which come to the community to do prevention activities. The opportunity of linking prevention with clinical services has been missed.	

#### Self-management support

Some health centres reported uptake of self-management activities, such as goal setting with clients and clients' families, however, these activities were not routinely documented (Table 7). One-to-one education was the most commonly mentioned approach for the delivery of patient education. Peer or group education was seldom used.

**Table 7:** Approaches used by health centres for promoting self-management

Approach	Number of health centres (N = 12)	
	
	**Used frequently**	**Used sometimes**
One to one education	10	2
Peer education sessions	1	4
Group sessions (eg diabetic or hypertensive clients)	1	5
Use of interpreters	3	5
Working with the family, not just the individual	2	10
Identification of barriers and challenges for individuals	3	7
Set goals with clients (eg weight loss, reduction of HbA1c)	4	6
Documentation of personal goals in the client files	0	5

Although acknowledging the importance of promoting self-management to individuals, families and groups in the community setting rather than only at the health centre, respondents cited that understaffing prevented them expanding their work in the community (Table 8).

**Table 8:** Examples of comments on self-management support

Self-management support	
**Weaknesses/threats (negative)**	**Strengths/opportunities (positive)**

"At the moment there is no health promotion so education around chronic disease is limited to within the health centre and understaffing is a problem. The situation would be improved if a nurse and a health worker could go out and educate in the community each week. People don't like coming to the clinic – it is necessary for the staff to get out and see people on the beach".	"A weight scale is provided at the clinic, and people come in and weigh themselves".
"There was an attempt to start up an exercise group in one of the rooms of the health centre, however, indemnity insurance rates make the idea impossible to implement".	"Sit down together and talk about the fact that a care plan would help. Set achievable goals with clients, eg their weights and blood pressure, then both sign agreement and agree on next visit. Give praise about what is going right. Keep messages positive. Review pathology and see the numbers dropping to reinforce behaviours which improve the condition".
	"People with hypertension and diabetes don't feel sick. So, it is necessary to talk with them and discuss illness. The staff encourage them to come in with their family so that other family members also understand the importance of taking medications etc".

#### Decision support

Clinical guidelines (e.g. CARPA standard treatment manual) [20] were universally distributed to centres to facilitate clinical decision-making, and most health centres reported the use of these guidelines in routine care. Involvement of specialists in primary care was mainly through conventional referrals, and visiting specialist services to health centres were perceived generally as not frequent enough to meet needs.

#### Delivery system design

As shown in Table 9, most health centres suffered from a shortage of staff, especially of AHWs and doctors. Only one health centre employed adequate (and ideal number of) AHWs in relation to the population size, and as a whole, health centres only employed about half (51%) of the number of AHWs specified as the ideal standard. Five communities had a resident doctor. Staffing levels were best for nurses, with 89% of the ideal level. Notably, two health centres (code F and K) were assessed as overstaffed by nurses (167% and 133% of the ideal respectively) but extremely understaffed by AHWs (no one employed), indicating the imbalance of the staff composition.

**Table 9:** Actual compared to ideal staffing level for each participating health centre

Community	population	AHWs			Nurses			Doctors		
				
		**actual**	**ideal**	**Actual as a % of ideal**	**actual**	**ideal**	**Actual as a % of ideal**	**actual**	**ideal**	**Actual as a % of ideal**
A	350	2	4.7	43%	1	1.8	56%	0.1	0.9	11%
B	850	1	4.3	23%	3	2.8	107%	0.6	1.1	55%
C	864	**2**	4.3	47%	2	2.9	69%	**0.2**	1.1	18%
D	1,500	**6**	6.0	100%	**2**	3.3	61%	**2**^§^	1.5	133%
E	480	**4**	4.8	83%	**2**	2.4	83%	0.4	0.8	50%
F	180	0	2.4	0%	2	1.2	167%	0^†^	0.5	0%
G	-- *	1	--	--	1	--	--	**2**^§^	--	--
H	1,100	**3**	5.5	55%	**4**	3.7	108%	1^§^	1.4	71%
I	700	**5**	7.0	71%	3	3.5	86%	1^§^	1.2	83%
J	560	**3**	5.6	54%	**3**	2.8	107%	1^§^	0.9	111%
K	300	0	4.0	0%	**2**	1.5	133%	0.7	0.8	88%
L	450	1	4.5	22%	1	2.3	43%	0.5	0.8	63%

Total ^‡^	7334	27	53.1	51%	25	28.2	89%	7.5	11	68%

* Population is not easily defined.

† Doctor had not visited the community F for the audited 3 month period due to industrial dispute.

‡ excluding the data from community G.

§health centres with resident doctors.

Numbers in bold denote having both male and female staff.

All centres reported that systems for collecting and reporting of pathology specimens and for dispensing medication were in place, which were also identified as strengths of the delivery system design (see Table 10). Other examples of good delivery system design included the provision of transport for clients to health centres, separate men's and women's clinic rooms, and available male and female health staff, specific roles and responsibility of team members for supporting the chronic disease program, and good teamwork characterised by effective communication (i.e. regular meetings) and coordination between team members for addressing issues related to chronic illness care. However, high staff turnover was perceived as a weakness with the delivery system, as it prevented clients from developing stable relationships with health centre staff and therefore discouraged their attendance at clinics. Other weaknesses in delivery system design included lack of staff training in health promotion and prevention and lack of specification of roles among team members with respect to coordination of chronic illness care.

**Table 10:** Examples of comments on delivery system design

Delivery system design	
**Weaknesses/threats (negative)**	**Strengths/opportunities (positive)**

"Irregularity of doctors is a big factor in this clinic. Sometimes there are none, and if there is, they are always changing – people don't like that – no one likes that, not in any community, people like to go to the same doctor, that doctor may not be very good, but people go with longer term relationship, with someone who knows them".	Clients are reminded by appointment cards (delivered by the driver the day before a visit is due). A list of clients is prepared and the driver goes to pick up everyone. Aboriginal Health Workers know if the person is at home or not. "People won't attend if not picked up – and it is a good thing in the heat to pick up the old people especially but also pick up the young people".
"From October to February a new nurse came every 5 weeks. People don't want to have anything to do with them. They don't know them. These staff never really got to know the place or the people and therefore were only partly effective".	The health centre has both male and female practitioners and consulting spaces. Well men's screening has been carried out since the arrival of a male Aboriginal health worker. Men use the back door for screening – a separate male entry so that they don't have to sit with the women. The Aboriginal health worker is thought of very well by his colleagues – "He is the backbone of men's health and shows up to work every day. The Yolngu* know that with him confidentiality is 100%".
"Annual diabetic eye checks are delivered by an external team located in nearby town. When people go to the town they have to wait up to 5 hours then they leave and don't wait and then don't get the service and have to wait another year".	A dosette system has been set up to increase medication compliance. The dosette boxes are filled at the health centre, then are delivered to people's homes and picked up by health centre staff.
The male AHW was working as a plumber and then heard via another male AHW that the health system was looking for a male AHW who was literate and numerate. He was trained but felt that the course didn't prepare him for working in a health centre. The course also contained little on chronic disease care.	"If someone has to have a fasting blood sample the health centre will open early to accommodate that person's needs on any day of the week. Samples are spun down if necessary, and put into the cold box. Courier picks up before mid-day each day – results fax back as soon as they are processed. The result goes through to GP's in-tray and he signs it as sighted. Then to doctor's out-tray (may have comments such as follow-up required). Nurse on call reads all results and files in Doctor's out-tray, and checks that action has been taken before they can be filed".
Health centre staff reported that a lack of training in health promotion, prevention or brief intervention was affecting the performance of staff in delivery of chronic illness care.	The registrar has been assigned the position of managing the chronic disease program. There are two team members who have a specific role for supporting chronic disease program.
The clinic manager has the assumed position of managing the chronic disease program. There is no team member who has a specific role or responsibility for the chronic disease program.	"When staff are regular there are weekly meetings and chronic disease issues are addressed, in broad terms i.e. recall, reminder and follow-up for due clients".
The Aboriginal Health Workers are across issues concerning chronic disease, but have no responsibility.	The health centre staff consider their team as cohesive – they have meetings every morning, they each go through the case loads, they discuss any recall reminders and they solve problems with systematic follow-ups.
Communication among team members may be an issue – internal meetings are supposed to be held monthly but these have been sporadic because of staff changes and if health workers are not there then it is not possible.	The recall and follow-up system is functioning well because of the team approach and everyone is aware of who is due to come, for what and who is responsible.
Chronic disease coordinator position is not recognised locally or by the system. The work between the doctor, nurses and Aboriginal workers is currently not well coordinated.	The health centre provides a place of work and the opportunity to work as a team. Administration officer is an active member of the team and coordinate care between the nurse, Aboriginal health worker and doctor. The doctor is a "good member of the team, good team player, cleans his own instrument, and gets them himself off the trolley."

* Aboriginal people inhabiting the north-eastern Arnhem Land of Australia

#### Clinical information systems

Computerised information systems were installed in 11 health centres, with four different clinical information software systems (see Table 11). The remaining centre used a paper-based information system only (see Figure 1). Recall systems were operational for eight health centres (6 computerised, 1 paper-based, and 1 with both). All centres had paper-based patient medical records, which were stored in filing cabinets and generally accessible.

**Table 11:** Computerised information systems in participating health centres

Computerised information systems	Description
CCTIS: Coordinated Care Trial Information System	First introduced to the Coordinated Care Trial sites (the Katherine West region and the Tiwi Islands) in December 1997, the CCTIS provides the facility for scheduling guideline services for individual clients, for identification of people due for scheduled services, and reminders to clinicians.
PCIS: Primary Care Information System	Funded by the NT health department, PCIS is a system evolving from the CCTIS. After the first version was piloted in 2002, the current version (3.2) is still under testing and validation process. The PCIS is expected to replace the CCTIS at the Tiwi Islands in the second half of 05/06.
Ferret	Ferret is a computer-based system introduced to the East Arnhem Land region by an Aboriginal Medical Service in 2000. It is used for client medical records and the chronic disease register.
Medical Director	Medical Director is a widely used clinical software system in Australia, which provides a simple to use prescription writing, medication, and electronic patient management system. It is estimated that 85% of GPs in Australia who have chosen to computerise their clinical practice use Medical Director [36].
Health *Connect*	The NT Health *Connect *trial was launched in October 2002 in the Katherine region to establish a suitable infrastructure that would facilitate secure messaging of consumer health summaries from a range of existing electronic information systems (eg CCTIS, PCIS and Ferret) used by different health service providers across the region [37].

Some health centres using the Coordinated Care Trial Information System (CCTIS) and Primary Care Information System (PCIS) expressed their concerns that the systems were too complex to be used as efficient tools that met clinical needs (Table 12). Other concerns generally appeared to be related to lack of IT maintenance support arrangement so that when things went wrong it was hard to have them fixed timely. Most information systems lacked the capacity (or were not easily used) to supply staff with population-based information on quality of chronic illness care. On the other hand, the piloted Health *Connect *project was perceived as an opportunity to transfer patient information across different information systems used in health centres, to accommodate the high mobility of Indigenous populations.

**Table 12:** Examples of comments on clinical information systems

Clinical information systems	
**Weaknesses/threats (negative)**	**Strengths/opportunities (positive)**

"Running paper and computer systems together is a problem and there is no choice in this". The health centre manager wishes they were on computer entirely as "not good to have both" – preferable to have one system because the dual system makes the process longer.	"The computerised system (Medical Director) is a convenient tool – the computer provides so many prompts and reminders eg if breastfeeding will give warning if medication is contraindicated; it can chart progress graphically (eg weight and BP); and the computer generates standard letters for specific appointments (eg optometrist or ophthalmologist for diabetic retinopathy patients)".
The system (CCTIS) wasn't designed for clinical needs. It takes too long to open and go through, and even to put in a diagnosis is complex and time-consuming. "Having to navigate through a maze of screens, backwards and forwards, to find information that is not collated, is counter productive and user unfriendly".	"The doctor comes Monday and Tuesday each week (and does one day per week of office work in Darwin for the health centre). He generates a list of follow-ups that are necessary and faxes it through to each staff member involved and follows up when he comes. This system works very well for staff and clients".
The new computer information system PCIS was introduced in December this year. The health centre has had the version 1 and 2 of PCIS and are now waiting for the version 3. The staff appear to have little confidence that version 3 will be better. Part of the problem is that the system has been designed around data collection needs rather than their immediate clinical needs – whereas they would be able to use population statistics in the future they want a system that is responsive to their daily needs – eg for recall etc. If it stays as is, then it just won't get used.	A project called Health *Connect *is being trialled in the region at the moment to see whether patient information can be transferred electronically between health service providers (community health centres and hospitals). In remote areas, population is highly mobile. "With Health *Connect *that information stays in the system wherever the person goes". The system will contain a recent medical event summary of patients, diagnostic results, pathology, x-rays and discharge summary.
There is no reporting on progress in chronic illness care. The health centre is only reporting on "the basics – the daily stats sheet". "It is more a record to provide evidence of the workload in the health centre".	

## Discussion

This study demonstrated the practical application of the Chronic Care Model concept for understanding primary care systems in relation to prevention and management of chronic illness in Australia's Indigenous communities. Participating health centres had both strengths and weaknesses in each of the system components. The status of health centre system development reflects both the availability of resources and the quality of management practices.

### Strengths and limitations of the study

The use of quantitative data in addition to qualitative data in this study enhanced our ability to assess the status of system development. The data may be subject to respondent bias, as staff interviewed may describe their health centre systems in a more favourable or critical light than warranted by actual conditions. However, with information on human, financial and material resource aspects of health centre systems, qualitative statements from health staff were assessed for consistency with quantitative data.

The emerging fields of chaos theory and the science of complex adaptive systems indicate health care systems are immensely complex [21,22]. The elements of health systems are changeable, the boundaries fuzzy, the relationships non-linear, and the behaviour emergent and sensitive to small changes. In order to describe health centre systems, this study broke them down into smaller components, and also only observed them at a single point in time. Efforts to describe systems at a single point in time using structured frameworks inevitably run the risk of oversimplification.

This study was based on 12 Aboriginal community health centres selected purposively from a pool of 53 health centres. The in-depth case study presents the particular situation on one health centre. Therefore, the findings are not necessarily generalisable to all health centres in the region. However, the findings do provide a general picture of how health centre systems become manifest within the framework of the CCM.

### Policy environment and health centre system development

Community health centres in the study had distinct areas of strength and weakness in each system component. These may be influenced by a variety of factors, such as different governance models, geographic locations, staff morale and conditions/situation of the community at the time of the survey. However, positive policy environment plays a fundamental role in driving and shaping community health centre development, especially through financing allocation and development and support of human resources [11].

#### Financing policy

Consistent financing allows health centres to invest in infrastructure and to employ adequate staff to support functioning systems. As demonstrated in this study, health centres able to provide separate men's and women's clinic rooms were regarded as having good delivery system design. There were a few health centres with a doctor resident in the community, which were also assessed as close to or reaching the ideal doctor level in relation to the population. Prominently, one health centre even had 100% of ideal staffing for AHWs. All of these centres had one feature in common – they had participated in the Aboriginal Coordinated Care Trials, which provided for community control of pooled funding from Commonwealth and NT governments [23]. This is likely to have contributed to the better staffing levels in these centres.

Following the Coordinated Care Trials, a new financing strategy was promoted for national implementation to improve Indigenous primary health care systems – the Primary Health Care Access Program (PHCAP). This program was to provide a mechanism for pooling of Commonwealth and State/Territory primary health care funds in designated local areas [24], and to redress the huge gap in Commonwealth funded Medicare expenditure per person for Indigenous Australians (whose expenditure was 37% of that for non-Indigenous people) [25,26]. The Commonwealth funding was to be based on a two- to four-fold multiple of average national Medicare health expenditure, and would have provided significant additional Commonwealth funding for Indigenous primary health care. However, implementation of PHCAP has been slower than initially planned, largely due to the challenges of securing agreement with all the partners (Federal Government, States and Territories and Community Controlled Health Sector) on financial, planning and implementation arrangements [27].

Financial incentives encouraging comprehensive and quality care have been introduced to Australian general practice through the Enhanced Primary Care (EPC) and Practice Incentive Programs (PIP) [28-30]. For example, incentive payments will be paid to reward practices for having a disease register, and GPs for completion of structured diabetes care (through Medicare rebates) [30]. However, only half of participating centres reported claiming for Medicare rebates using EPC items. There is one absolute criterion to be an eligible practice for the PIP: the practice must be fully accredited [24]. In this study, half of participating health centres were accredited. When feasible, health centres should be supported to gain accreditation status to secure additional funding for their system development. Another important issue noted in this study is that, while Medicare rebates are used to reward the individual GP in private general practice, that money seldom goes into the hands of doctors who serve Indigenous community health centres in salaried positions (see Table 5). This indicates that nationwide practitioner-based incentives oriented at general practice may require modification for the purpose of improving Indigenous primary care systems.

#### Development of human resources

This study highlights the shortage of AHWs across community health centres. This situation results from a number of reasons, including inadequate funding for AHW positions and difficulties in recruitment and retention of AHW staff. The influence of the quality of AHW – nurse relationships on the number of AHWs actually working in the health centres has been highlighted by work in Central Australia [31]. Jackson and colleagues have reported that sound and respectful working and interpersonal relationships between AHWs and nurses can be achieved by enhancing workplace equity and skill sharing between the two groups [32]. Currently, national health workforce-to-Indigenous population ratio targets are being developed, which is an important step towards effectively allocating and targeting human resources for Indigenous primary health care [33].

Our qualitative findings indicate that lack of training and support were significant barriers for AHWs in developing their role in chronic illness care. In addition, poor documentation of activities appears to contribute to under-recognition of AHWs' work. Community health centres need to improve management practices to enforce the role and contribution of AHWs in chronic illness care, as illustrated in the Aboriginal Coordinated Care Trials [34]. At the policy level, there will be an injection of $100 million funding to expand primary health care workforce in the Northern Territory from mid 2008, with a specific goal to recruit and train more AHWs for providing health promotion and preventive services outside community health centres (Dr Christine Connors, personal communication).

The complexity of service delivery in Indigenous communities requires high level teamwork, particularly effective communication and coordination among primary care team members. Many remote community health centres are staffed primarily by nurses and AHWs and supported by visiting doctors. A patient's journey from having an HbA1c test to seeing a doctor involves many steps. Usually a nurse or AHW orders an HbA1c test and collects the blood sample when a patient visits the health centre. Then the blood sample is couriered to a designated pathology laboratory. After the result returns to the health centre, the nurse may arrange for it to be reviewed by a doctor who visits the clinic one day per fortnight. The doctor may request that patient attends the clinic on the day when the doctor visits next time. The consultation occurs only when the patient shows up on that day. Any step missed leads to a failure in follow up of the patient. As tasks are divided among primary care team members, the need for effective communication and coordination is even greater. The importance of interactions and coordination among primary care team members has been highlighted in a recently published systematic review; it found organisational interventions promoting interconnections between and co-evolution of individual team members achieved greater improvements in diabetes patient outcomes [35].

### Implications for policy and practice

This study has important potential to contribute to improving readiness and capability of many clinicians in adopting systems thinking in their busy day-to-day care. Based on practical examples presented in the paper, it is clear that six system components of the Chronic Care Model manifest themselves in every facet of community health centre operations (internal and external), and delineate connections between individual patients, health staff, families and communities. Uptake of systems thinking will help shift the focus from the individual clinicians to the systems in which they work, and is more likely to effectively improve patient outcomes.

## Conclusion

Using concrete examples, this study translates the concept of the Chronic Care Model (and associated systems view) into practical application in Australian Indigenous primary care settings. This approach proved to be useful in understanding the quality of primary care systems for prevention and management of chronic illness. This study demonstrates that Indigenous community health centre systems had distinct areas of strength and weakness

The identified strengths of current systems suggest that remote Indigenous health centres are generally keeping abreast with the international practice in developing chronic care oriented systems. This should encourage policy makers to continue investment in Indigenous primary care systems and to create a positive policy environment for driving and shaping system development. On the other hand, identified weaknesses point to the need for refinement of current primary health care systems, not only by increasing financial, staffing and other resources, but also by seeking to implement improved and innovative management practices. These should aim to maximise the effectiveness and efficiency of primary care systems, and, most importantly, to enhance interaction between health care providers and patients.

## Competing interests

The authors declare that they have no competing interests.

## Authors' contributions

DS participated in developing the study design, performed the statistical analyses, and drafted the manuscript. RB conceived and designed the study, supervised the data collection and analyses, provided a major role in revising the manuscript. MD and AS developed questionnaires and performed the data collection. JC, GR, CC and TW participated in the development of study design, editing and revising the manuscript. All authors contributed to, have read and approved the final manuscript.

## Pre-publication history

The pre-publication history for this paper can be accessed here:


